# Network Meta-Analytical Investigations of the Performance of HIV Combination Prevention Strategies for Indigenous Populations

**DOI:** 10.3390/v17091247

**Published:** 2025-09-16

**Authors:** Marcos Jessé Abrahão Silva, Rebecca Lobato Marinho, Daniele Melo Sardinha, Diego Rafael Lima Batista, Luiza Raquel Tapajós Figueira, Tamires de Nazaré Soares, Keitty Anne Silva Neves, Aloma Mapinik Suruí, Manuella Nunes Colaço, Vinicius dos Santos Peniche, Ligia Regina Franco Sansigolo Kerr, Sebastião Kauã de Sousa Bispo, Ana Judith Pires Garcia, Carl Kendall, Luana Nepomuceno Gondim Costa Lima

**Affiliations:** 1 Postgraduate Program in Parasite Biology in the Amazon (PPGBPA), State University of Pará (UEPA), Belém 66087-662, PA, Brazil; rebeccamarinho28@gmail.com (R.L.M.); danielle-vianna20@hotmail.com (D.M.S.); lrtfigueira@gmail.com (L.R.T.F.); tamiresenfsoares@hotmail.com (T.d.N.S.); contatoknatj@gmail.com (K.A.S.N.); 2 School of Health Sciences, State University of the Amazonas (UEA), Manaus 69065-001, AM, Brazil; rafaelarcanjo18@gmail.com; 3 Special Indigenous Health District-Vilhena Indigenous Health Secretariat-SESAI, Porto Velho 76804-444, PA, Brazil; alomasurui2@gmail.com; 4 Pará State Secretariat for Indigenous Peoples (SEPI), Belém 66015-040, PA, Brazil; manuella.colaco@outlook.com; 5 Law Department, Damásio University, São Paulo 01503-001, SP, Brazil; penichevinicius@gmail.com; 6 Faculty of Medicine, Federal University of Ceara (UFC), Fortaleza 60430-160, CE, Brazil; ligiakerr@gmail.com; 7 Evandro Chagas Institute (IEC), Ananindeua 67030-000, PA, Brazil; enfkauabispo@gmail.com (S.K.d.S.B.); anaquaresma@iec.gov.br (A.J.P.G.); 8 Department of Social, Behavioral and Population Sciences, Tulane University School of Public Health and Tropical Medicine, New Orleans, LA 70112, USA; carl.kendall@gmail.com

**Keywords:** HIV prevention, indigenous populations, network meta-analysis, combination prevention, home-based counseling, SUCRA, intercultural health

## Abstract

Background: Indigenous populations worldwide face a disproportionate burden of HIV due to structural inequities, cultural marginalization, and limited access to health services. Despite growing recognition of the need for culturally adapted responses, the effectiveness of combination HIV prevention strategies in these communities remains underexplored. Objectives: This study aimed to evaluate and compare the effectiveness of multiple HIV prevention strategies among Indigenous populations using a systematic review and network meta-analysis (NMA), to inform equity-oriented public health interventions. Methods: Following PRISMA-NMA 2020 guidelines, a comprehensive literature search was conducted across four databases (PubMed, SciELO, LILACS, Science Direct) for quantitative studies published between January 2000 and June 2025. Eligible studies evaluated HIV prevention interventions among Indigenous populations and reported risk or odds ratios. A frequentist NMA model was used to calculate effect estimates (OR, 95% CI) and SUCRA rankings for seven types of interventions, combining biomedical, behavioral, and structural approaches. Results: Four high-to-moderate quality studies enclosing 4523 participants were included. The most effective intervention was home-based counseling and testing for HIV, followed by medical consultation combined with HIV testing. Standalone testing, while effective, was significantly less impactful than when combined with culturally sensitive educational strategies. Information-only strategies showed the least efficacy. The SUCRA analysis ranked home-based testing highest (45.17%), highlighting the importance of decentralization, community participation, and intercultural mediation. Conclusions: Culturally adapted combination prevention strategies—especially those integrating home-based testing and counseling—are more effective than isolated biomedical interventions in Indigenous populations. These findings reinforce the urgent need for participatory, context-driven public health responses that center Indigenous knowledge, reduce stigma, and expand equitable access to HIV care and prevention.

## 1. Introduction

Human immunodeficiency virus (HIV) remains one of the foremost global public health challenges. Over recent decades, significant progress has been made in the prevention, diagnosis, and treatment of HIV infection, including expanded access to testing, antiretroviral therapy (ART), and pre-exposure prophylaxis (PrEP) [1]. However, these advancements have not benefited all populations equally. Among the most vulnerable groups, Indigenous peoples continue to face historical and structural barriers that hinder access to healthcare services and prevention resources [2,3].

In Brazil, official data indicates a concerning increase in HIV detection rates among Indigenous communities in certain regions, particularly within the Legal Amazon, despite their relatively small share of the national population [4]. These findings highlight not only the spread of the epidemic among Indigenous peoples, but also the inadequacy of conventional prevention strategies when applied to sociocultural contexts that are often overlooked by universal health policies [5].

HIV prevention among Indigenous populations demands integrated approaches that consider language, customs, territorial identity, and specific worldviews. Recent studies have shown that strategies grounded in community participation, bilingual education, and intercultural mediation yield more substantial results in terms of adherence to care practices and stigma reduction [6,7]. Furthermore, evaluating traditional knowledge systems and promoting Indigenous leadership in the design of interventions have been identified as key elements for ensuring the effectiveness of such strategies [8,9].

In recent years, the concept of combination HIV prevention has been widely promoted by international organizations and ministries of health as an approach that integrates biomedical (such as PrEP and regular testing), behavioral (such as condom use), and structural (such as addressing stigma) measures. This strategy is recognized for its flexibility and adaptability to different sociocultural contexts, and is particularly relevant for indigenous populations, whose knowledge and care practices are often incompatible with conventional biomedical models [10].

Combination HIV prevention is a strategy that uses a combination of different biomedical, behavioral, and structural interventions to reduce the risk of new HIV infections. This approach considers the individual, social, and cultural needs and specificities of individuals and their contexts, offering multiple prevention options that can be used alone or in combination. Among the tools included in combination prevention are the use of male and female condoms, regular HIV testing, PrEP, post-exposure prophylaxis (PEP), antiretroviral treatment for people with HIV, immunizations, as well as behavioral interventions and structural changes that help reduce the risk of transmission. The goal is to enable each person to consider the strategies that best fit their reality, increasing the effectiveness of prevention in a sustainable and comprehensive manner [11,12].

Although national and international guidelines recognize the need to adapt HIV prevention actions to Indigenous contexts, systematic evidence on the actual effectiveness of these strategies across different regions and cultures remains scarce. The available literature is fragmented, composed of studies with heterogeneous designs, diverse populations, and varied interventions, making it difficult to develop recommendations based on robust evidence [2,13].

Furthermore, much of the research on HIV among Indigenous people is qualitative or descriptive, lacking quantitative indicators that allow for standardized comparisons of different interventions. In this context, Network Meta-Analysis (NMA) emerges as a useful tool, as it allows for the synthesis and comparison of multiple interventions even when they have not been directly compared with each other in all studies. By bringing together direct and indirect comparisons, NMA provides a probabilistic hierarchy of the most effective strategies, offering objective support for policy and clinical decisions [14].

In this context, the present meta-analysis aims to gather and critically assess scientific evidence on the efficacy and effectiveness of HIV prevention strategies implemented among Indigenous populations between the years 2000 and 2025. This initiative seeks not only to map the outcomes of these interventions but also to identify their limitations and strengths, thereby contributing to the development of more equitable and culturally sensitive public policies.

## 2. Materials and Methods

### 2.1. Study Design and Settings

This meta-analysis was conducted based on the PRISMA-NMA (Preferred Reporting Items for Systematic Reviews and Meta-Analyses Involving a Network Meta-analysis) guidelines, aiming to ensure transparency, reproducibility, and methodological rigor throughout all stages, from the research question formulation to the final synthesis of findings. The PRISMA-NMA checklist was fully followed, including protocol registration, eligibility criteria, screening process, data extraction, and assessment of the quality of the included studies [15]. This meta-analysis was registered in PROSPERO under the code CRD420251084973.

Network meta-analysis (NMA) offers significant advantages over traditional meta-analyses, which typically compare only two interventions at a time. One of the greatest advantages of NMA is the ability to simultaneously compare multiple interventions in a single analysis. Furthermore, it integrates both direct and indirect evidence, broadening the available evidence base. This is especially relevant in clinical settings where multiple intervention options are available, allowing for a broader and more comprehensive evaluation of alternatives, as is the current case with HIV prevention [16].

### 2.2. Search Strategy

The literature search was conducted in PubMed, SciELO, LILACS (via BVS), and Science Direct between June and July 2025. The following descriptors were used, combined with Boolean operators: (“HIV” OR “AIDS”) AND (“combined prevention” OR “PrEP “ OR “condom use” OR “counseling”) AND (“Indigenous populations” OR “Native communities”) AND (“efficacy” OR “effectiveness”). Studies published between January 2000 and June 2025, in Portuguese, Spanish, or English, with full-text access, were included. The strategy was adapted according to the specificities of each database.

### 2.3. Eligibility Criteria

Eligible studies were those published between January 2000 and June 2025 that addressed HIV prevention strategies among Indigenous populations and provided quantitative outcomes (odds ratios, risk ratios, or equivalent measures). Qualitative studies, systematic reviews, case reports, and studies involving mixed populations without stratification for Indigenous peoples were excluded. No language restrictions were applied, and only full-text studies were included [17,18]. Systematic reviews, qualitative studies, case reports, editorials, as well as studies with mixed populations without specific stratification for indigenous people were excluded. Study screening was conducted in two phases by two independent reviewers (MJAS and RLM). Initially, titles and abstracts were reviewed, followed by full-text reading. Discrepancies were resolved by consensus, with a third reviewer (DMS) consulted when necessary, in accordance with the guidelines of the Cochrane Handbook for Systematic Reviews of Interventions [19].

### 2.4. Study Selection and Data Extraction

Study selection and data extraction were performed through two stages by two independent reviewers (MJAS and RLM). In this stage, full texts were analyzed for eligibility. Disagreements were resolved by consensus, with a third reviewer (DMS) consulted when necessary.

Data extracted from each study included: author and year of publication, country of study, study population, study type, prevention strategy evaluation, measure of effect (such as odds ratio, risk ratio, proportion, or prevalence), main results, and the platform from which the article was obtained. Data were organized in Excel spreadsheets and analyzed using Python software v.3.13.7.

### 2.5. Methodological Quality Assessment and Risk of Bias

Methodological quality was assessed using a matrix adapted from instruments developed by the GRADE system. Six dimensions were evaluated: clarity of study design, population definition, alignment between objectives and methods, outcome measurement, bias control, and relevance to public policies. Each study was rated as having high, moderate, or low methodological quality, based on criteria outlined by Guyatt et al. (2011) [20].

The reliability of the evidence was judged according to GRADE criteria, with greater weight assigned to studies with low risk of bias, internal consistency, and direct applicability. The limitations of individual studies were explicitly presented, and the strength of the evidence was discussed considering these limitations, enhancing transparency and the utility of the findings [20,21].

### 2.6. Ranking Analysis

In this NMA, we used CINeMA (Confidence in Network Meta-Analysis) website to assess the confidence in the evidence obtained (https://cinema.ispm.unibe.ch/; accessed on 20 May 2025). CINeMA is based on pre-established criteria that consider six essential domains: risk of bias, inconsistency, imprecision, incoherence, selective publication, and methodology of the included studies. Each domain was systematically assessed to classify the quality of the evidence from high to very low confidence. This approach allowed for a comprehensive and transparent assessment of the robustness of the NMA results, facilitating the interpretation of the findings and helping to identify potential limitations and biases that could influence the conclusions. The analyses were conducted following the recommendations in the literature, ensuring methodological rigor and reliability in data synthesis [22].

### 2.7. Statistical Analysis

For studies that reported odds ratios (OR) or risk ratios (RR) with confidence intervals, the data were standardized to OR. Risk ratios were converted to OR using the formula: OR ≈ RR/[1 − P0 + (P0 × RR)], where P0 represents the incidence of the outcome in the control group, estimated at 20% for comparative standardization [23].

The network meta-analysis (NMA) was conducted using a random-effects model, given the small number of studies, and point estimates of OR and 95% CI were calculated for each direct and indirect comparison. The consistency of the network was verified visually and by comparing the direct and indirect effects. The interventions were ranked according to the P-score (frequentist), which ranges from 0 (least effective) to 1 (most effective), and represents the probability of an intervention being the best among those evaluated [24].

The comparison network was visualized using a graph with nodes and three direct connections (A–B, A–C, B–C), enabling the calculation of the cross-effects matrix. All data organization and visualizations were performed using Python (pandas, matplotlib, network), with manual cross-validation of the extracted data [25].

The SUCRA was used to assess the ranking of preventive intervention options evaluated based on the studies included in this meta-analysis. The SUCRA metric is a statistical measure used primarily in network meta-analyses to provide a probabilistic ranking of treatments or interventions. It estimates the likelihood that a particular treatment is among the best options compared to others in the network, based on the available evidence. However, the SUCRA does not provide information about the absolute effect size or the certainty of the evidence; it only indicates relative ranking probabilities [26,27].

## 3. Results

### 3.1. Characteristics of Included Studies

**Figure 1:**
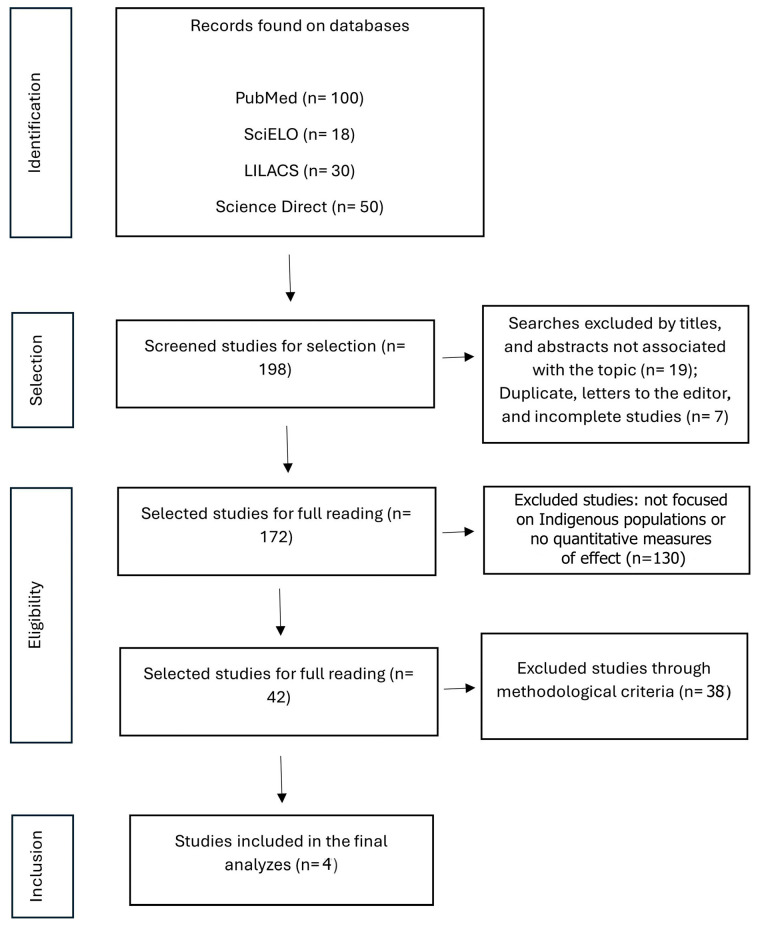
PRISMA Flowchart of the stages of selection and eligibility criteria processes on search, databases for this meta-analysis data material extraction.

Figure 1 shows the PRISMA flowchart, which details the phases of study identification, screening, eligibility, and inclusion in the review. Initially, many records were identified from the four databases consulted (*n* = 198). Next, duplicate studies (*n* = 7) and those not associated with the topic (*n* = 19) were removed, and, subsequently, a total of 130 excluded records comprised studies that either (a) did not specifically address Indigenous populations, (b) did not provide quantitative outcomes (e.g., only qualitative or descriptive results). After reading the abstracts and full texts, 38 studies were excluded from the analysis this time by independent peer review, resulting in only 4 studies meeting all eligibility criteria and being included in the network meta-analysis. Four studies met the eligibility criteria and were included in this meta-analysis, encompassing a variety of HIV prevention strategies applied to Indigenous populations in different Latin American and global contexts. These interventions included biomedical approaches (such as Testing and Care for HIV), and behavioral measures (such as home-based counseling and testing for HIV) and structural types (receiving enough information about STIs) [28,29,30,31].

In addition to outcome synthesis, a methodological quality assessment was performed using adapted GRADE tools. Among the 4 studies reviewed, a total of 3 studies were classified as high quality (75%), and 1 as moderate quality. The strongest domains across studies were Defined Population and Relevance to Public Policy (75% rated as “high” each one), whereas the methodological clarity varied substantially (Table 1) [28,29,30,31].

### 3.2. Network Structure and Data Analyzed

**Figure 2:**
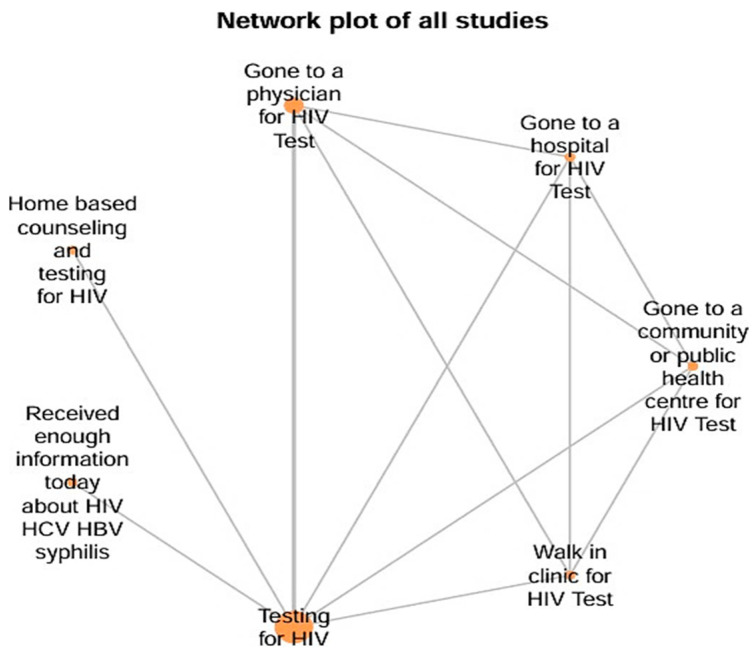
Network Plot of Interventions Strategies for this Meta-analysis.

The network structure consisted of 7 intervention nodes: Testing for HIV (A), Gone to a physician for HIV Test (B), Gone to a hospital for HIV Test (C), Gone to a community or public health center for HIV Test (D), Walk-in clinic for HIV Test (E), Home-based counseling and testing for HIV (F) and Received enough information today about HIV/HCV/HBV/syphilis (G). A total of 4523 subjects were included in this meta-analysis with an interconnected network and 12 direct pairwise comparisons, 21 possible pairwise comparisons and 3 two-arm studies. The lines connecting the nodes represent direct comparisons made by the included studies, while the absence of connections indicates indirect comparisons possible through the network model. The distribution of nodes and connections demonstrates the centrality of testing (A), connected to several other interventions, and reveals how community strategies, especially testing with home counseling (F), occupied a prominent position in terms of comparability. Thus, the figure visually explains how the different contexts of testing and counseling were interrelated in the analyzed studies, serving as a basis for probabilistic comparisons of effectiveness (Figure 2).

Based on direct and indirect comparisons between the studies, it was possible to estimate inconsistency data in Supplementary Material Table S1. The confidence domains of this meta-analysis were systematically assessed using CINeMA, classifying the quality of evidence as high for all included studies, and were described in Supplementary Material Table S2. Data Summary of included studies were performed on Table 2 [28,29,30,31].

### 3.3. Main Results

**Figure 3:**
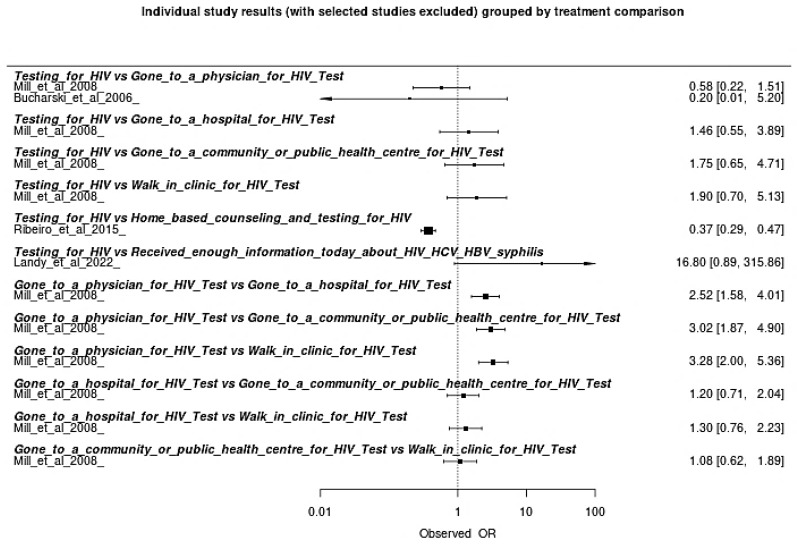
Forest Plot of Individual Analysis of Efficacy within Prevention HIV Strategies on this Meta-Analysis [28,29,30,31].

A forest plot was constructed for individual analysis comparing the interventions analyzed for indigenous populations based on data from the included articles (Figure 3). The most effective strategy was the combination of home counseling with HIV testing, which outperformed the other options in terms of effectiveness (more favorable OR). The second-best performance was the combination of doctor visits and HIV testing, representing a mixed strategy of health access and biomedical prevention. HIV testing alone was considered effective, but less impactful than when combined with educational or behavioral strategies. Strategies based solely on access to information about HIV/HCV/HBV/syphilis or in institutional settings further from the community (such as hospitals or outpatient clinics) had lower effectiveness, suggesting lower adherence or impact with these approaches.

**Figure 4:**
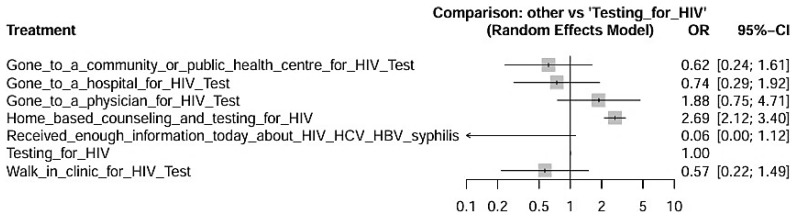
Forest Plot showing Measures Effects with 95% Confidence Intervals for HIV prevention interventions in Indigenous populations with Testing for HIV as a reference factor for comparisons.

Figure 4 details the effects of the interventions compared to HIV testing alone, acting as the reference intervention. The findings showed that home counseling and testing showed a statistically significant superior effect compared to testing alone, with a confidence interval that does not cross the null line (OR > 1), indicating statistically significant greater effectiveness. Visiting a doctor for testing also showed better performance compared to testing alone, although with a smaller effect size. Strategies such as visiting hospitals or public/community clinics did not show statistically significant differences compared to testing alone—suggesting that the location and format of the intervention directly impact its effectiveness. The intervention “Receiving information about STIs” had the lowest relative effectiveness, with odds ratios close to or below neutrality, indicating low effectiveness alone.

The SUCRA ranking, which expresses the probability of an intervention being the most effective among those evaluated, confirmed this trend: Home-based counseling and testing for HIV + testing for HIV obtained the highest score (45.17%), followed by Gone to a physician for HIV Test + Testing for HIV (31.60%), and finally, Only Testing for HIV alone with 16.80% (Table 3). These data demonstrate that, although Testing for HIV is an effective tool, its implementation alone is less effective than when combined with educational, and behavioral strategies. Due to the low number of articles, it was not possible to present the Funnel Plot of this network meta-analysis, with the risk of presenting substantial risks in identifying publication biases in the studies. It should provide a concise and precise description of the experimental results, their interpretation, as well as the experimental conclusions that can be drawn.

## 4. Discussion

This network meta-analysis is the first to quantitatively synthesize the effectiveness of combined HIV prevention strategies specifically in Indigenous populations, a group historically neglected in global and national public health policymaking. By integrating data from studies conducted in diverse cultural and geographic contexts—such as Brazil, Canada, and Australia—the results demonstrate not only the heterogeneity of interventions but also convergent patterns that reinforce the importance of culturally sensitive and territorially appropriate approaches [32].

The combined HIV prevention approach is particularly relevant for Indigenous populations because isolated biomedical strategies often fail to address the complex social, cultural, and structural factors that influence health in these communities. Many Indigenous populations face multiple barriers such as geographic isolation, language differences, mistrust of healthcare systems due to historical and ongoing oppression, and limited healthcare resources. These barriers reduce the effectiveness and accessibility of purely biomedical interventions like PrEP or treatment alone. Culturally grounded, contextual interventions acknowledge and integrate Indigenous worldviews, traditions, and community strengths, which can improve the acceptability, relevance, and sustainability of prevention efforts [33,34].

Structural determinants such as poverty, discrimination, and limited healthcare access are fundamental drivers of HIV disparities in Indigenous communities. Poverty often limits access to basic needs like stable housing, nutritious food, and healthcare services, creating environments where vulnerability to HIV is heightened. Discrimination—whether racial, ethnic, or cultural—manifests in social stigma, exclusion from healthcare, and reduced trust in medical institutions, which discourages Indigenous peoples from seeking testing, prevention, and treatment services. Limited healthcare access, due to geographic remoteness, lack of culturally sensitive care, or systemic barriers, further exacerbates these issues by reducing timely diagnosis and continuity of care [35,36].

These structural factors interact synergistically, reinforcing cycles of marginalization and health inequity. For example, unstable housing and poverty increase exposure to risk behaviors and reduce adherence to HIV treatment. Stigma within healthcare settings leads to avoidance of services, worsening health outcomes. Moreover, policies and systemic inequities rooted in colonialism and racism perpetuate these disparities by maintaining social and economic exclusion. Addressing HIV in Indigenous populations requires tackling these upstream determinants through multi-level interventions that improve social conditions, dismantle discrimination, and enhance equitable healthcare access [37].

In this sense, the main emerging evidence of this present study was the superiority of home counseling strategies combined with HIV testing, which showed the largest relative effects in direct and indirect comparisons, in addition to the highest score in the SUCRA ranking. This finding suggests that the territorialization of preventive actions—that is, their direct insertion into communities—can act as a catalyst for trust, adherence, and engagement among the Indigenous population. This effectiveness can be explained not only by facilitated access, but also by the appreciation of interpersonal relationships and the collective care logic present in many Indigenous groups [38].

In contrast, isolated testing strategies, while effective, had less impact when disconnected from educational and community actions. This highlights that the biomedical dimension of prevention alone is insufficient to generate lasting changes in contexts marked by institutional distrust, language barriers, stigma, and structural inequality. Interventions rooted in community-based approaches and intercultural dialog demonstrated positive outcomes, especially when integrated with culturally sensitive education and health promotion. Previous studies indicated that bilingual communication, Indigenous leadership engagement, and respect for traditional knowledge were critical to enhancing acceptability and adherence to preventive practices [39,40,41,42].

In our present study, the culturally adapted combination prevention strategies were those that integrated biomedical (testing), behavioral (counseling, health education), and structural (community participation, territorialization, cultural sensitivity) dimensions. Strategies based solely on technical information, without cultural contextualization, were the least effective (SUCRA 3.28%). Culturally adapted strategies were those that brought prevention into Indigenous territory (home, community) and incorporated counseling, community participation, and respect for local sociocultural practices, overcoming isolated biomedical logic. These strategies included home-based counseling and HIV testing, medical consultation combined with HIV testing, and testing combined with culturally sensitive educational strategies [28,29,30,31].

Despite the relevance of the present synthesis, it is important to acknowledge the intrinsic heterogeneity among the included studies and the very limited number of eligible articles. Only four studies, with diverse methodological designs, contexts, and intervention strategies, met the inclusion criteria. This scarcity of quantitative evidence on HIV prevention in Indigenous populations reflects a broader structural gap in global research, where these groups remain historically underrepresented [43,44]. The small number of studies inevitably limits the statistical power of this network meta-analysis and precludes more refined subgroup or sensitivity analyses [26]. Furthermore, heterogeneity in terms of cultural settings, health systems, and intervention delivery methods should be interpreted as both a limitation and a signal of the complex realities faced by Indigenous communities. Therefore, while the convergent pattern observed—particularly the superiority of home-based counseling combined with HIV testing—offers meaningful insights, these findings must be viewed with caution and considered as a starting point for future, more robust investigations specifically designed to address the diversity of Indigenous contexts.

We also consider some characteristic points of our present study, which used predefined concepts from the articles included in this review for analysis, which were: Home-based counseling and testing (interventions conducted in participants’ homes, typically using rapid HIV tests, accompanied by pre- and post-test counseling sessions); Medical consultation combined with testing (in-person consultation physicians in clinical or community settings, followed by HIV testing); Information-only strategies (provision of written or oral educational materials without individualized counseling). All these strategies mentioned above from the studies were culturally adapted for their implementation. In Brazil, home-based counseling was adapted through bilingual communication and community health workers from Indigenous backgrounds. In Canada, Métis-led initiatives emphasized community-driven pilot projects and collective decision-making. In Australia, HIV testing campaigns were delivered with respect for local customs and confidentiality [28,29,30,31].

Furthermore, the poor performance of interventions based solely on providing information on HIV, HCV, HBV, and syphilis highlights that decontextualized information, even when technically accurate, does not automatically translate into behavioral transformation or adherence. Technical knowledge needs to be translated and appropriated within the meanings of Indigenous groups, which reinforces the urgency of bilingual interventions, with visual resources, and with the participation of local leaders—as already proposed by Nicholson et al. (2021) and Kemp et al. (2024) [7,8]. Behavioral interventions such as condom promotion and educational workshops have led to increased HIV knowledge and changes in sexual behavior among adolescents and young adults [45].

Testing conducted in conventional medical settings, such as clinics or hospitals, achieved intermediate performance. Although this type of intervention represents an improvement over isolated testing, it still faces logistical and cultural obstacles. Several authors (e.g., Walters et al., 2020; Craig Rushing, 2012) point out that the centralization of healthcare in urban or institutionalized services represents a symbolic and practical barrier to access for Indigenous peoples, especially those in remote regions. The consistency of findings across studies from different countries and designs reinforces the robustness of the evidence produced. Although the number of studies included in this analysis is limited, the findings are consistent and robust, indicating a promising path for redesigning HIV prevention actions in Indigenous contexts [9,39,46,47].

Another important dimension revealed by this review concerns the methodological quality of the included studies. The greatest consistency of findings was observed in studies classified as high-quality, which demonstrated greater coherence between objectives and methods, as well as relevance for public policymaking [48]. On the other hand, lower-quality studies presented flaws in bias control and population definition. These findings point to the urgent need for investment in robust, longitudinally structured research that considers the specificities of Indigenous peoples from methodological design to analysis and feedback.

The small number of studies included in this meta-analysis reflects the critical gap in disaggregated and specific data on indigenous populations in the field of HIV prevention. With such a small evidence base, the ability to detect differences across interventions is limited, and the results should therefore be interpreted with caution. Despite the heterogeneity of contexts, the identified patterns indicate that strategies that combine testing, counseling, and cultural territorialization are consistent across countries. This consistency across different contexts reinforces the argument that cultural adaptation of interventions is more than desirable—it is essential for their effectiveness [14,49].

Moreover, our analysis was limited by the level of detail provided in the included studies, which often aggregated participants without further cultural stratification. These characteristic compromises the accuracy of the interpretation of the results, since different Indigenous groups can present significant variations in social, cultural, and behavioral factors related to HIV prevention. We explicitly emphasize that the results presented here may not apply equally to all Indigenous groups, reinforcing the need for future research that considers cultural and regional diversity for a more contextualized and effective evaluation of HIV prevention strategies [50].

Besides that, the included studies were heterogeneous in terms of design and methodology. While all provided quantitative indicators, some were descriptive or cross-sectional in nature, and none were randomized controlled trials. This variability reduces the overall strength of the evidence and increases the risk that confounding factors influenced the reported outcomes. In particular, the absence of longitudinal designs limits the capacity to assess causal relationships or the sustainability of intervention effects over time [51]. Another limitation here was that the scope of available data was constrained by the lack of disaggregation within studies. Most studies did not stratify outcomes by gender, age, or specific Indigenous subgroups, which prevents a more nuanced understanding of how interventions may perform across different populations. Given the cultural and contextual diversity among Indigenous communities worldwide, this lack of granularity may obscure important differences in intervention effectiveness. We also acknowledge challenges in scaling such interventions, including workforce shortages, funding limitations, and structural barriers [9,52]. Moreover, because of the limited number of eligible articles, it was not possible to formally evaluate small-study effects or publication bias.

Finally, our findings align with the principle of health equity, which proposes treating the unequal unequally to promote social justice. In the case of Indigenous populations, this implies recognizing that solutions must emerge with and from communities, respecting their knowledge, ways of life, and forms of care. Such approaches not only produce better health outcomes but also promote processes of self-determination and health sovereignty [53]. To this end, it is recommended to build intersectoral, sustainable strategies developed in dialog with local leaders, Indigenous professionals, and intercultural mediators [54].

In summary, this network meta-analysis makes a unique contribution to the field of combination HIV prevention in Indigenous contexts, offering robust and hierarchical evidence that can inform more effective public policies that are sensitive to local realities. We recommend that future prevention programs be planned in partnership with Indigenous leaders, utilize flexible testing and education formats, and be sustainably funded to ensure continuity and long-term impact [55].

Then, the results of this review expand understanding of the contextualized effectiveness of HIV prevention interventions in Indigenous populations, highlighting that choosing the most effective strategy should not be based solely on clinical evidence alone, but also on criteria of acceptability, community participation, and sociocultural relevance [6,56]. The hierarchy of effectiveness revealed by SUCRA reinforces the need to reorient public HIV policies toward models centered on equity, territorialization, and the self-determination of Indigenous peoples. Therefore, this review not only provides scientific evidence for improving interventions but also reaffirms the ethical commitment to social justice, health sovereignty, and the effective inclusion of Indigenous populations in public policies to combat HIV [7,52].

These disparities highlight the importance of considering the methodological rigor of each study when synthesizing evidence and developing culturally appropriate and effective public health interventions for Indigenous populations. However, given the diversity of study designs and cultural contexts, it underscores the need for more standardized and rigorous approaches in future research within this field [57]. However, significant challenges remain, such as the lack of disaggregated data and structural barriers that hinder continued access to health services. Addressing HIV in Indigenous contexts requires a committed approach centered on equity, with public policies developed through participatory processes and aligned with the lived realities of these communities [9,56,58].

Ethical considerations regarding research and implementation of HIV preventive measures involving Indigenous communities must also be considered, as it is essential to prioritize Indigenous leadership to ensure culturally appropriate and community-focused approaches. Obtaining informed consent must go beyond formalities to ensure that participants fully understand the purpose, methods, and potential impacts of the research. Respect for traditional knowledge and practices is crucial, recognizing the sovereignty of Indigenous peoples over their cultural heritage. Researchers must actively avoid exploitative practices to prevent historical abuses and foster relationships of trust, justice, and reciprocity, strengthening the legitimacy and effectiveness of preventive interventions in the Indigenous context. They must promote equitable partnerships, transparency, and benefit-sharing, thus fostering trust and ethical integrity throughout the research process. Respect for traditional knowledge is essential to valuing and protecting Indigenous cultural practices, recognizing their legitimacy and contribution to collective health. These ethical principles ensure that health research and strategies do not perpetuate inequalities and contribute to the empowerment and well-being of the communities involved [59,60,61].

## 5. Conclusions

This network meta-analysis demonstrated, based on quantitative and comparative evidence, that combination HIV prevention strategies are more effective among Indigenous populations when they incorporate educational and counseling interventions, especially in home and community settings. The superiority of these interventions lies not only in their biomedical framework, but also in their ability to promote trust, acceptance, and belonging through cultural sensitivity and active community participation. Culturally adapted strategies that integrate education, counseling, and testing showed greater adherence and impact.

The best performance was observed in educational approaches involving counseling with testing, followed by actions involving healthcare combined with testing for HIV. Isolated testing strategies, while useful, proved less effective, and those focused solely on providing information had even more limited performance. These results confirm that decontextualized interventions have little power to transform behavior, especially in historically marginalized areas with a strong link between cultural identity and health practices. More than adapting existing models, it is necessary to build health responses that respect the knowledge, languages, and ways of life of Indigenous peoples.

## Figures and Tables

**Table 1:** Methodological Quality Assessment of the 4 Included Studies (Adapted from GRADE system).

Study	Methodological Clarity	Clearly Defined Population	Coherence Between Objectives and Methods	Measurable Out-Comes	Bias Control	Relevance to Public Policy	Overall Quality
Mill et al. (2008) [31]	High	Moderate	High	High	Low	High	High
Bucharski et al. (2006) [28]	High	High	High	High	Moderate	High	High
Ribeiro et al. (2015) [29]	Moderate	High	High	Low	High	High	High
Landy et al. (2022) [30]	Moderate	High	Low	High	High	Moderate	Moderate

**Table 2:** Data Summary of HIV Prevention Strategies in Indigenous Populations.

Author/Year	Country	Population	Type of Study	Evaluated Strategy	Individuals (*n*)	Search Database
Mill et al. (2008) [31]	Australia	Indigenous people from Aboriginal ancestry of the Anangu Pitjantjatjara Lands	Cross-sectional	Testing and care	205	PubMed
Bucharski et al. (2006) [28]	Canada	Canadian Aboriginal ancestry (First Nations, Metis)	Descriptive study	Testing and care	7	Science Direct
Ribeiro et al. (2015) [29]	Brazil	From Brazilian Amazon Region.	Cross-sectional	Testing and Home-based counseling and testing	1861	SciELO
Landy et al. (2022) [30]	Canada	Indigenous people within a Métis cultural context.	Cross-sectional	Testing and sexual health education	26	PubMed

**Table 3:** SUCRA Ranking Evaluation for the Nodes of Meta-Analysis.

Intervention	SUCRA Score (%)	SUCRA Ranking
Home-based counseling and testing for HIV	45.17 [2.38; 857.01]	1st
Gone to a physician for HIV Test	31.60 [1.46; 683.46]	2nd
Testing for HIV	16.80 [0.89; 315.86]	3rd
Gone to a hospital for HIV Test	12.45 [0.57; 272.01]	4th
Gone to a community or public health center for HIV Test	10.37 [0.47; 227.14]	5th
Walk-in clinic for HIV test	9.57 [0.44; 209.90]	6th
Received enough information today about HIV HCV HBV syphilis	3.28 [2.00; 5.36]	7th

## Data Availability

The original contributions presented in the study are included in the article. Further inquiries can be directed at the corresponding author.

## Supplementary Materials

The following supporting information can be downloaded at: https://www.mdpi.com/article/10.3390/v17091247/s1, Table S1: Summary Data on Inconsistency of Studies Included in this Network Meta-analysis, Table S2: CINeMA Analysis for Included Studies in this Meta-analysis.
