# Supplementary material for: Network Meta-Analytical Investigations of the Performance of HIV Combination Prevention Strategies for Indigenous Populations
Source: Viruses. 2025 Sep 16;17(9):1247. doi: 10.3390/v17091247 (PMC12474319; doi:10.3390/v17091247)
Supplement: Supplementary file 1 [file viruses-17-01247-s001.zip › Supplementary Material S2.pdf]

Supplementary Table S2. CINeMA Analysis for Included Studies in this Meta-analysis.

Comparison	Number of studies	Within-study bias	Reporting bias	Indirectness	Imprecision	Heterogeneity	Incoherence	Confidence rating	Reason(s) for downgrading
Gone to a community or public health centre for HIV Test:Gone to a hospital for HIV Test	1	No concerns	Low risk	No concerns	Some concerns	Some concerns	No concerns	High	[]
Gone to a community or public health centre for HIV Test:Gone to a physician for HIV Test	1	No concerns	Low risk	No concerns	No concerns	Major concerns	No concerns	High	[]
Gone to a community or public health centre for HIV Test:Testing for HIV	1	No concerns	Low risk	No concerns	Some concerns	Some concerns	No concerns	High	[]
Gone to a community or public health centre for HIV Test:Walk-in clinic for HIV Test	1	No concerns	Low risk	No concerns	No concerns	Major concerns	No concerns	High	[]
Gone to a hospital for HIV Test:Gone to a physician for HIV Test	1	No concerns	Low risk	No concerns	No concerns	Major concerns	No concerns	High	[]
Gone to a hospital for HIV Test:Testing for HIV	1	No concerns	Low risk	No concerns	Some concerns	Some concerns	No concerns	High	[]
Gone to a hospital for HIV Test:Walk-in clinic for HIV Test	1	No concerns	Low risk	No concerns	Some concerns	Some concerns	No concerns	High	[]

Gone to a physician for HIV Test:Testing for HIV	2	No concerns	Low risk	No concerns	Some concerns	Some concerns	No concerns	High	[]
Gone to a physician for HIV Test:Walk-in clinic for HIV Test	1	No concerns	Low risk	No concerns	No concerns	Major concerns	No concerns	High	[]
Home-based counseling and testing for HIV:Testing for HIV	1	No concerns	Low risk	No concerns	No concerns	Major concerns	No concerns	High	[]
Received enough information today about HIV/HCV/HBV/ syphilis:Testing for HIV	1	No concerns	Low risk	No concerns	Some concerns	Some concerns	No concerns	High	[]
Testing for HIV:Walk-in clinic for HIV Test	1	No concerns	Low risk	No concerns	Some concerns	Some concerns	No concerns	High	[]
Gone to a community or public health centre for HIV Test:Home-based counseling and testing for HIV	0	No concerns	Low risk	No concerns	No concerns	Major concerns	No concerns	High	[]
Gone to a community or public health centre for HIV Test:Received enough information today about HIV/HCV/HBV/ syphilis	0	No concerns	Low risk	No concerns	Major concerns	No concerns	No concerns	High	[]
Gone to a hospital for HIV Test:Home-based counseling and testing for HIV	0	No concerns	Low risk	No concerns	No concerns	Major concerns	No concerns	High	[]

Gone to a hospital for HIV Test:Received enough information today about HIV/HCV/HBV/ syphilis	0	No concerns	Low risk	No concerns	Some concerns	Some concerns	No concerns	High	[]
Gone to a physician for HIV Test:Home-based counseling and testing for HIV	0	No concerns	Low risk	No concerns	Some concerns	Some concerns	No concerns	High	[]
Gone to a physician for HIV Test:Received enough information today about HIV/HCV/HBV/ syphilis	0	No concerns	Low risk	No concerns	No concerns	Major concerns	No concerns	High	[]
Home-based counseling and testing for HIV:Received enough information today about HIV/HCV/HBV/ syphilis	0	No concerns	Low risk	No concerns	No concerns	Major concerns	No concerns	High	[]
Home-based counseling and testing for HIV:Walk-in clinic for HIV Test	0	No concerns	Low risk	No concerns	No concerns	Major concerns	No concerns	High	[]
Received enough information today about HIV/HCV/HBV/ syphilis:Walk-in clinic for HIV Test	0	No concerns	Low risk	No concerns	Major concerns	No concerns	No concerns	High	[]
