# Supplementary material for: Network Meta-Analytical Investigations of the Performance of HIV Combination Prevention Strategies for Indigenous Populations
Source: Viruses. 2025 Sep 16;17(9):1247. doi: 10.3390/v17091247 (PMC12474319; doi:10.3390/v17091247)
Supplement: Supplementary file 1 [file viruses-17-01247-s001.zip › Table S1.pdf]

Supplementary Table S1. Summary Data on Inconsistency of Studies Included in this Network Meta-analysis.

	Comparison	No.Studies	NMA	Direct	Indirect	Difference	Diff_95CI_lower	Diff_95CI_upper	pValue
1	Gone_to_a_community_or_public_health_centre_for_HIV_Test:Gone_to_a_hospital_for_HIV_Test	1	-0,183138217	-0,183138217	NA	NA	NA	NA	NA
2	Gone_to_a_community_or_public_health_centre_for_HIV_Test:Gone_to_a_physician_for_HIV_Test	1	-1,114485082	-1,106809056	-4,08109406	2,974285005	-6,53419698	12,48276699	0,539821
3	Gone_to_a_community_or_public_health_centre_for_HIV_Test:Home_based_counseling_and_testing_for_HIV	0	-1,471680629	NA	-1,471680629	NA	NA	NA	NA
4	Gone_to_a_community_or_public_health_centre_for_HIV_Test:Received_enough_information_today_about_HIV_HCV_HBV_syphilis	0	2,338720072	NA	2,338720072	NA	NA	NA	NA
5	Gone_to_a_community_or_public_health_centre_for_HIV_Test:Testing_for_HIV	1	-0,482949132	-0,559615788	0,694066353	-1,253682141	-5,261574469	2,754210187	0,539821
6	Gone_to_a_community_or_public_health_centre_for_HIV_Test:Walk_in_clinic_for_HIV_Test	1	0,08035663	0,08035663	NA	NA	NA	NA	NA
7	Gone_to_a_hospital_for_HIV_Test:Gone_to_a_physician_for_HIV_Test	1	-0,931346866	-0,923670839	-3,673009919	2,74933908	-6,040014015	11,53869217	0,539821
8	Gone_to_a_hospital_for_HIV_Test:Home_based_counseling_and_testing_for_HIV	0	-1,288542412	NA	-1,288542412	NA	NA	NA	NA
9	Gone_to_a_hospital_for_HIV_Test:Received_enough_information_today_about_HIV_HCV_HBV_syphilis	0	2,521858289	NA	2,521858289	NA	NA	NA	NA
10	Gone_to_a_hospital_for_HIV_Test:Testing_for_HIV	1	-0,299810915	-0,376477571	0,854682511	-1,231160082	-5,167051716	2,704731551	0,539821

11	Gone_to_a_hospital_for_HIV_Test:Walk_in_clinic_for_HIV_Test	1	0,263494847	0,263494847	NA	NA	NA	NA	NA
12	Gone_to_a_physician_for_HIV_Test:Home_based_counseling_and_testing_for_HIV	0	-0,357195546	NA	-0,357195546	NA	NA	NA	NA
13	Gone_to_a_physician_for_HIV_Test:Received_enough_information_today_about_HIV_HCV_HBV_syphilis	0	3,453205155	NA	3,453205155	NA	NA	NA	NA
14	Gone_to_a_physician_for_HIV_Test:Testing_for_HIV	2	0,631535951	0,631535951	NA	NA	NA	NA	NA
15	Gone_to_a_physician_for_HIV_Test:Walk_in_clinic_for_HIV_Test	1	1,194841713	1,187165686	4,274876814	-3,087711128	-12,95880471	6,783382458	0,539821
16	Home_based_counseling_and_testing_for_HIV:Received_enough_information_today_about_HIV_HCV_HBV_syphilis	0	3,810400701	NA	3,810400701	NA	NA	NA	NA
17	Home_based_counseling_and_testing_for_HIV:Testing_for_HIV	1	0,988731497	0,988731497	NA	NA	NA	NA	NA
18	Home_based_counseling_and_testing_for_HIV:Walk_in_clinic_for_HIV_Test	0	1,552037259	NA	1,552037259	NA	NA	NA	NA
19	Received_enough_information_today_about_HIV_HCV_HBV_syphilis:Testing_for_HIV	1	-2,821669204	-2,821669204	NA	NA	NA	NA	NA
20	Received_enough_information_today_about_HIV_HCV_HBV_syphilis:Walk_in_clinic_for_HIV_Test	0	-2,258363442	NA	-2,258363442	NA	NA	NA	NA
21	Walk_in_clinic_for_HIV_Test:Testing_for_HIV	1	-0,563305762	-0,639972418	0,625066184	-1,265038603	-5,309236365	2,77915916	0,539821
